# Investigation of self-treatment with lysergic acid diethylamide and psilocybin mushrooms: Findings from the Global Drug Survey 2020

**DOI:** 10.1177/02698811231158245

**Published:** 2023-03-06

**Authors:** Emma I Kopra, Jason A Ferris, Adam R Winstock, Kim PC Kuypers, Allan H Young, James J Rucker

**Affiliations:** 1 Department of Psychological Medicine, Institute of Psychiatry, Psychology and Neuroscience, King’s College London, London, UK; 2 Centre for Health Services Research, Faculty of Medicine, The University of Queensland, Brisbane, QLD, Australia; 3 Institute of Epidemiology and Health Care, University College London, London, UK; 4 Global Drug Survey Ltd, London, UK; 5 Department of Neuropsychology and Psychopharmacology, Faculty of Psychology and Neuroscience, Maastricht University, Maastricht, Netherlands; 6 South London and Maudsley NHS Foundation Trust, London, UK

**Keywords:** Psychedelics, LSD, psilocybin, self-treatment, mental health

## Abstract

**Background:**

Growing numbers of people are using psychedelics for personal psychotherapy outside clinical settings, but research on such use is scarce.

**Aims:**

This study investigated the patterns of use, self-reported outcomes and outcome predictors of psychedelic ‘self-treatment’ of mental health conditions or specific worries/concerns in life.

**Methods:**

We use data from the Global Drug Survey 2020, a large online survey on drug use collected between November 2019 and February 2020. In all, 3364 respondents reported their self-treatment experiences with lysergic acid diethylamide (*N* = 1996) or psilocybin mushrooms (*N* = 1368). The primary outcome of interest was the 17-item self-treatment outcome scale, items reflecting aspects of well-being, psychiatric symptoms, social-emotional skills, and health behaviours.

**Results:**

Positive changes were observed across all 17 outcome items, with the strongest benefits on items related to insight and mood. Negative effects were reported by 22.5% of respondents. High intensity of psychedelic experience, seeking advice before treatment, treating with psilocybin mushrooms and treating post-traumatic stress disorder were associated with higher scores on the self-treatment outcome scale after averaging values across all 17 items. Younger age, high intensity of experience and treating with LSD were associated with increased number of negative outcomes.

**Conclusions:**

This study brings important insights into self-treatment practices with psychedelics in a large international sample. Outcomes were generally favourable, but negative effects appeared more frequent than in clinical settings. Our findings can help inform safe practices of psychedelic use in the community, and inspire clinical research. Future research can be improved with utilisation of prospective designs and additional predictive variables.

## Introduction

Psychedelics have been used for healing purposes since prehistoric times in some cultures (Carod-Artal, 2015; Guerra-Doce, 2015). However, more widespread use, as well as clinical research on psychedelics in the West, did not start until the mid-20th century, following the discovery of lysergic acid diethylamide (LSD)’s psychoactive properties in 1943 (Hofmann and Ott, 1980), and the isolation and synthesis of psilocybin from ‘magic mushrooms’ in the following decade (Hofmann et al., 1959). Despite psychedelics’ eventual prohibition by the UN Convention on Psychotropic Substances in 1971, usage has continued underground ever since. Millions of people around the world take psychedelics every year, most commonly in informal settings, but increasingly under the supervision of underground therapists (Sessa and Fischer, 2015) or in retreat or therapy centres in regions where psychedelics are less strictly regulated (Rucker and Young, 2021).

With the recent resurgence of scientific research with psychedelics (Goodwin et al., 2022a; Rucker et al., 2016; Rucker et al., 2022; Weston et al., 2020), there is evidence that the use of these drugs in the community has also slowly increased over the past decade (Australian Institute of Health and Welfare, 2020; GOV.UK, 2019; Winstock et al., 2021; Yockey et al., 2020). Specifically, there are increasing accounts of people aiming to self-treat their conditions with psychedelics (Butler et al., 2023; Mason and Kuypers, 2018; Matzopoulos et al., 2021; Soares et al., 2022), also reflected by increased demand for clinicians specialising to provide support for such clients, and community-led group integration circles (Pilecki et al., 2021). This trend is likely to continue as the public’s awareness of psychedelics’ therapeutic potential grows, even though it is likely to be some years until they may be approved as medicines (McCartney et al., 2022; Muthukumaraswamy et al., 2022). Even if licensed psychedelic therapy was to become widely accessible, self-treatment may be preferred by some who are sceptical of westernised psychedelic therapy models or those simply preferring the freedom of choosing their dose, environment and company.

The user’s mindset and individual characteristics (‘set’) and physical and social environment (‘setting’) are known to be essential in determining the nature of psychedelic experiences. Therefore, these aspects are given close attention in modern clinical trials, from inclusion criteria and design of the dosing room to the therapeutic support provided before, during and after the psychedelic experience (Carhart-Harris et al., 2018). Consequently, the generally favourable outcomes observed from these trials (Andersen et al., 2021) may not be generalisable to the safety and efficacy of taking psychedelics outside such contexts. It is reassuring that even naturalistic psychedelic use has been found to be associated with a relatively low risk of acute harms (Kopra et al., 2022a; Kopra et al., 2022b), as well as with sustained increases in a range of well-being and mental health-related aspects in numerous prospective and retrospective surveys (Agin-Liebes et al., 2021; Garcia-Romeu et al., 2019; Garcia-Romeu et al., 2020; Mans et al., 2021; Mason et al., 2019; Mason et al., 2020; Spriggs et al., 2021; Zeifman et al., 2020). However, individual responses to psychedelics vary widely and notably, there is a lack of research on psychedelic use specifically intended for the self-treatment of emotional distress and psychiatric concerns. To our knowledge, the only investigation to date on psychedelic self-treatment of mental health conditions by Mason and Kuypers (2018) found overall outcomes as more favourable compared to treatments offered by medical professionals; however, the study lacked any further detail regarding treatment patterns and specific outcomes.

Examining motivations for self-treatment, how people self-treat, with what outcomes, and how specific treatment patterns and user characteristics may predict those outcomes, may inform harm reduction advice and guide safer use among the public. Crucially, although reported outcomes from naturalistic use seem mostly positive, psychedelics’ ability to bring up unprocessed trauma or grief may cause heightened psychological risks in clinical populations (Johnson et al., 2008; Watts et al., 2017). Psychedelic-induced acute challenging experiences and emotions are common and often considered part of the therapeutic process (Carbonaro et al., 2016; Gashi et al., 2021; Shaw et al., 2022). However, without adequate interpersonal support and supervision, these experiences may be destabilising and cause longer-lasting distress (Johnson et al., 2008; Watts et al., 2017). Furthermore, investigation on self-treatment can also help fill information gaps left by current, early phase clinical trials that rely on small sample sizes, apply strict inclusion criteria, and utilise relatively fixed, homogeneous protocols regarding the key aspects of the therapy (Carhart-Harris et al., 2022; McCartney et al., 2022). These study designs draw from early literature on the best-known practices of delivering psychedelic therapy safely and effectively, but have left alternative approaches little investigated and, for instance, excluded many patient groups often due to safety concerns or in order to minimise sample heterogeneity.

This study investigates experiences of people reporting self-treatment of mental health conditions or specific personal worries with LSD or psilocybin-containing mushrooms, in a large international sample of Global Drug Survey (GDS) respondents. Specifically, we explore the patterns of self-treatment and experienced negative and positive outcomes and aim to identify the potential predictors of these outcomes regarding demographics, patterns of treatment (substance used, intensity of psychedelic experience and obtaining advice beforehand), and specific condition or indication treated.

## Methods

### Design

The GDS is an annual, anonymous and encrypted online survey on substance use. It is advertised on social networking sites in collaboration with media partners and harm reduction organisations. It has to date collected data from over 900,000 people, with data used to create free harm reduction tools and academic publications (Winstock et al., 2022). Available to anyone with access to the URL and restricted to those 16 years of age or older, this survey reaches large numbers of respondents engaging in both common and rare drug consumption practices and societally stigmatised behaviours. The purpose of the survey is to capture drug consumption experiences from people who use drugs including sentinel drug using populations allowing the early detection of new drug trends and practices before they reach the general population. As a non-probability sample, the survey is not representative of wider drug using populations but allows in-depth analyses exploring patterns of use across different groups and the relationships between different patterns of use and risk of harms and perceived benefits.

GDS 2020 was launched on the 7th of November 2019 and was available until the 5th of February 2020, in 16 languages. Participants were not remunerated. Full details about the survey design and recruitment, including related discussion on the survey’s utility can be found elsewhere (Barratt et al., 2017). Multi-institutional ethical approval was obtained from the King’s College London (11671/001), University of Queensland (No: 2017001452) and The University of New South Wales (HREC HC17769) Research Ethics Committees. Ethics review boards required that participants were allowed to skip questions and leave empty responses if they did not want to complete specific items. Access to the relevant sections of the GDS 2020 dataset (demographic data and sections on psychedelics) were obtained through a data sharing agreement with the GDS.

### Measures

#### Patterns of self-treatment

A wide range of demographic information was collected including age and gender, followed by a drug screen and detailed sections on use of the most common drugs for those who had used these in the last 12 months. At the end of the *psychedelic* section, respondents were asked whether they have ever used psychedelics (with an example list being presented: LSD, Magic Mushrooms^1^, Ketamine, MDMA, Peyote, Kambo, DMT, 5-MEO DMT, Ayahuasca or Ibogaine) with the specific intention of improving general mental health and well-being, managing a diagnosed psychiatric condition, and/or to address a specific worry or concern in their life (these three options not being mutually exclusive). Respondents indicating self-treatment of a diagnosed condition and/or a specific worry/concern within the past 12 months were then directed to an optional section enquiring more about these experiences.

To minimise the length of the survey and the associated time burden, respondents were asked to select which condition or experience they were *mainly* trying to treat from a list of 19 (including ‘Other’; Supplemental Table S1); and to select those psychedelics they had self-treated with within the past 12 months. Respondents then indicated the single substance they had found most useful and were instructed to answer all subsequent questions based on their self-treatment experiences with this substance.

Further questions about patterns of self-treatment included the number of times the substance was used for self-treatment purposes in the past 12 months; the intensity of the psychedelic experience relative to their chosen dose (5 response options: intense psychedelic experience; moderate psychedelic experience; mild psychedelic experience; no psychedelic experience but other acute effects; or no experience or effects at all; see Supplemental Materials); and whether respondents had obtained advice or information before using the substance for self-treatment. Respondents who indicated receiving information relating to use were asked to select the source(s) of information from nine options: a doctor, a therapist, a website, online forum, social media/news, a friend/partner/family member, a local psychedelic society, a book or other (not further specified).

#### 17-Item self-treatment outcome scale

Respondents reported on changes they had observed as a consequence of their self-treatment across 17 different aspects of well-being, psychiatric symptoms, social-emotional skills and health behaviours. Each item was rated on a 7-point scale, the two opposing values being −3 ‘strong negative consequences’ to +3 ‘strongly positive consequences’; 0 was considered ‘no change’, and respondents could also indicate not applicable (N/A). The items were created by the GDS research team, based on discussions with a group of psychedelic researchers, clinicians involved in psychedelic treatment delivery and psychiatrists; and a review of the literature. No psychometric testing was used for the construction of the scale, that is validity of the items was not established.

Respondents with a score of +2 or +3 on at least one item indicated the time-to-onset (relative to a first dose) of noticing most of the *positive effects* from 6 options (less than 12 h, 12–24 h, 1–2 days, 3–7 days, greater than 1 week, 1 month or more); and indicated how long most of these positive effects lasted by picking one of 12 options (less than 12 h to >6 months).

#### 10-Item Negative Outcome Scale

Respondents were also asked whether they had noticed any negative consequences from their self-treatment experiences. Those indicating ‘yes’ rated the occurrence of 10 different negative outcomes, on a four-option ordinal scale (none, mild, moderate and severe). Those having experienced negative outcomes also indicated the time-to-onset of noticing any negative effects from five options (less than 12 h, 12–24 h, 1–2 days, 3–7 days, more than 1 week from dosing); and how long most of the negative effects lasted from 12 options (less than 12 h to >6 months). Those reporting negative effects were also asked whether they had sought emergency medical treatment following self-treatment (yes, once; yes, more than once; no, never).

### Data pre-processing

#### Sample selection

As respondents were asked to report on their self-treatment experiences based on the substance they had found most useful (in case they had self-treated with several substances), this investigation was restricted to those respondents indicating either LSD or psilocybin mushrooms as the substance found most useful. These substances were judged suitable to be analysed together in the same investigation due to current evidence of their similar subjective effects and pharmacological and therapeutic mechanisms of action (Dos Santos et al., 2021; Holze et al., 2022), similar psychedelic therapy models applied in research, and preliminary evidence of comparable therapeutic outcomes (Leger and Unterwald, 2022).

#### 17-Item self-treatment outcome scale

Mean scores of the 17 outcome items for each participant were calculated by averaging all values with non-missing data (maximum of 17), with possible values ranging from −3 (strongly negative) to +3 (strongly positive outcomes). N/A responses were coded as missing values and therefore also excluded from the calculations. Cronbach’s alpha for the 17 items was excellent, α = 0.91.

#### 10-Item negative outcome scale

Negative outcomes were rated on an ordinal scale, and therefore could not be treated as continuous data. While an ordinal regression model could be applied for predicting responses to a single ordinally rated dependent variable, it is not possible to create any summary statistic of a multiple-item scale which would retain the ordinal nature of the data. Therefore, to create a regression model predicting overall prevalence of negative outcomes across all items, the items were first transformed into binary variables, indicating No/Yes presence of any severity of a negative outcome. In essence, ‘None’ responses were recoded as ‘0’ (No), and ‘mild’, ‘moderate’ and ‘severe’ as ‘1’ (Yes). Zeros were also coded for all items of those respondents who had earlier indicated not experiencing any negative outcomes, and who therefore were not presented with the 10-item list. Sum scores were then calculated for each respondent, creating a variable reflecting the total number of (mild-to-severe) negative outcomes with a minimum of 0 and maximum of 10. Those with no response to the question on whether negative outcomes were experienced, were excluded from these analyses. In turn, all cases indicating presence of negative outcomes were included in the analyses, regardless of any missing values on subsequent individual outcome items.

### Data analysis

Descriptive statistics with tables and graphs were created for demographics, patterns of self-treatment, and positive and negative outcomes, including responses to individual items on both outcome scales as well as time-to-onset and duration of outcomes. All percentages reported for descriptive statistics are valid percentages; that is, calculated from the numbers of respondents responding to a given question, with missing values excluded.

To investigate associations between predictive variables and scores on the 17-item outcome scale, a multiple linear regression (ordinary least squares (OLS) regression) with the mean outcome score as the dependent variable was conducted. Predictor variables included in the model were as follows: age (continuous), gender identity (hereinafter referred to as ‘gender’; Male/Female/Other), substance used (LSD/psilocybin mushrooms), seeking advice (Yes/No), intensity of experience (5 categories) and condition treated (13 categories). The initial 19 categories of ‘condition treated’ were reduced to 13 due to low counts in six categories each with 0.5% or less of respondents’ valid responses (see Supplemental Table S1). Specifically, ‘Cancer-related distress’ and ‘Mental health distress associated to cancer diagnosis’ were grouped together with ‘Distress associated with another medical disorder’, and the category renamed ‘Distress associated with a medical condition’. ‘Overeating (obesity)’, ‘To increase appetite’ and ‘Chronic pain’ were grouped together with the category ‘Other’. One category, ‘Treating cancer itself’, had a count of zero (i.e. no respondents indicated treating cancer) and was ignored.

Ordinal regression model was conducted to investigate associations between predictive variables and the duration of most positive outcomes (among those respondents with a score of +2 or +3 on at least one item on the 17-item self-treatment outcome scale). A test for proportional odds assumption of ordinal regression was undertaken and if violated, multinomial logistic regression model was adopted. For the purposes of this analysis, to ease interpretation of data and avoid too small *N*s within groups resulting in low power, the original 12 categories of the dependent variable were reduced to three; up to 7 days; 1 –4 weeks; and 1 month or more. Predictive variables included in the model were the same as in the multiple linear regression model.

Negative binomial regression was conducted to investigate associations between predictive variables and the number of the 10 negative outcomes experienced; negative binomial regression was used instead of Poisson regression to account for any overdispersion. Predictive variables included were the same as in the multiple linear regression model.

Similarly to analysis on duration of positive outcomes, ordinal regression model was conducted to investigate associations between predictive variables and the duration of most negative outcomes (among those respondents indicating at least some negative outcomes). Again, in case test for proportional odds assumption was violated, multinomial logistic regression model was adopted instead. The original 12 categories of the dependent variables were reduced to three, similarly to the analysis on duration of positive outcomes (up to 7 days, 1–4 weeks, and 1 month or more). Predictive variables included were the same as in the previous models.

The focus of analysis in this paper was only exploring associations between main effects and the outcomes; as such, no interaction terms were included in any of the regression analyses. Complete case analysis was used instead of imputation in all models, due to missingness (dropouts) likely occurring at random given the design of our study (cross-sectional survey); acceptable rates of missing data observed and the exploratory nature of our study (Hughes et al., 2019; Jakobsen et al., 2017). To correct for multiple tests conducted, *p* value was adjusted based on the number of factors of the predictive variables (21), resulting in a *p* value threshold of 0.05/21 = 0.002.

## Results

### Sample characteristics

**Figure 1.**
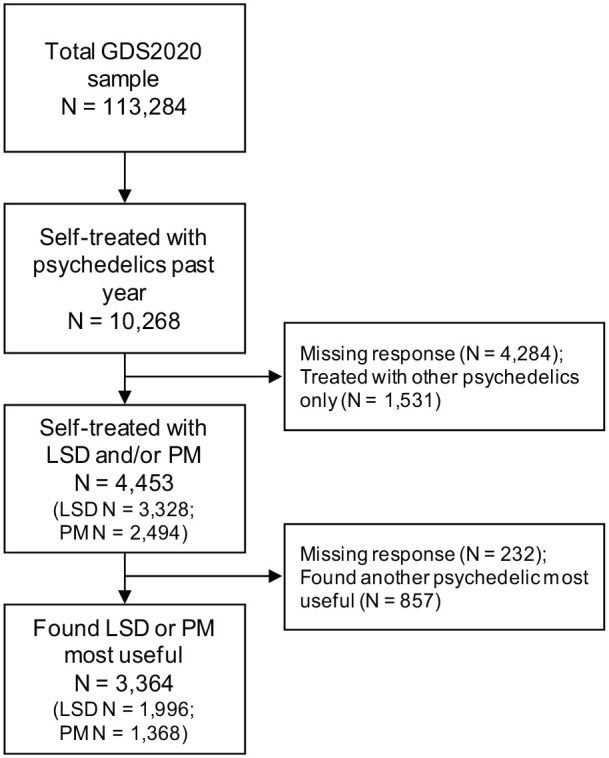
Response flow chart. LSD: lysergic acid diethylamide; PM: psilocybin mushrooms.

A response flow chart in Figure 1 demonstrates how the sample for the present investigation was obtained. A total of 113,284 participants took part in GDS 2020, of which 10,268 (valid % = 13.8) reported having self-treated their psychiatric condition or a specific worry with psychedelics within the past year. Of these, 3328 and 2494 respondents, respectively, indicated having at least used LSD and psilocybin mushrooms for this purpose in the past year (55.6% and 41.7% of the 5984 respondents reporting on substances used for self-treatment); and 1996 (35.4%) and 1368 (24.3%) indicated LSD and psilocybin mushrooms as the substance they found most useful. This resulted in a sample of *N* = 3364 for the final analyses.

The demographic characteristics of this sample are presented in Supplemental Table S2. The sample was predominantly young (mean = 25.4, standard deviation (SD) = 8.5), of male gender (*N* = 2422; 72.0%) and of white ethnicity (*N* = 2679; 80.3%). Majority of participants (*N* = 1899; 56.5%) reported having been diagnosed with one or more mental health conditions during their lifetime.

Of those having used both LSD and psilocybin mushrooms for self-treatment, 40.0% (*N* = 515) indicated LSD and 40.7% (*N* = 524) psilocybin mushrooms as their most preferred substance for self-treatment, indicating roughly equal preference between the substances among these respondents.

Descriptive statistics about patterns of treatment is shown in Table 1.

**Table 1.** Patterns of self-treatment with LSD and psilocybin mushrooms.

	*N*	Valid %
Substance used	3364	
LSD	1996	59.3
Psilocybin mushrooms	1368	40.7
Primary condition/problem treated	3327	
Depression	1338	40.2
Anxiety	666	20
Relationship problem	308	9.3
Trauma	164	4.9
Alcohol or other SUD	152	4.6
PTSD	118	3.5
Bipolar	66	2
Bereavement	63	1.9
Distress associated with a medical condition	48	1.4
OCD	27	0.8
Anorexia/bulimia	23	0.7
Psychosis	19	0.6
Other	335	10.1
[Missing]	[37]	
Intensity of experience	3228	
Intense psychedelic experience	1562	48.4
Moderate psychedelics experience	1175	36.4
Mild psychedelic experience	368	11.4
No psychedelic experience but other acute effects	102	3.2
No experience or effects at all	21	0.7
[Missing]	[136]	
Obtaining information beforehand	3104	
No	616	19.8
Yes* (from where:)	2488	80.2
Website	1656	53.4
Online forum	1522	49
Friend/partner/family member	1101	35.5
Book	811	26.1
Social media/news	590	19
Local psychedelic society	361	11.6
Therapist	123	4
Doctor	67	2.2
Other	259	8.3
[Missing]	[260]	
Frequency of treating in the past 12 months	3234	
1	863	26.7
2–3	1085	33.5
4–10	775	24
>10	511	15.8
[Missing]	[130]	
Used recreationally in the past 12 months	3241	
Yes	2405	74.2
No	836	25.8
[Missing]	[123]	
Types of help accessed for the treated condition in the past 12 months*	3364	
Mindfulness	1667	49.6
Seen a psychiatrist	1154	34.3
And used medication	739	22
Not received medication	415	12.3
Psychological talking therapies	944	28.1
Other counselling services	502	14.9
Seen a psychotherapist	501	14.9
Online support	462	13.7
Seen a family therapist	152	4.5
Acupuncture	115	3.4

* Able to select more than one.

LSD: lysergic acid diethylamide; OCD: obsessive compulsive disorder; PTSD: post-traumatic stress disorder; SUD: substance use disorder.

### Treatment outcomes: descriptives

#### 17-Item self-treatment outcome scale

Descriptive statistics in this subsection are based on 3145 cases who had one or more valid responses on the 17-item outcome scale. The occurrence of missing values (including N/As) across all responses on the 17 items was 3.36%; and the percentage of respondents with 5 or more missing values was 3.31%. We therefore concluded missing values occurring at non-random were unlikely to affect the results substantially. To further confirm this, an additional sensitivity analysis was performed for the OLS regression predicting scores on the 17-item scale, among those respondents only with less than 5 missing values or N/As. This produced similar results to the regression conducted with the full sample (Supplemental Table S3). No cases or averages were therefore excluded from the main analyses regardless of the number of valid responses on the outcome items.

On a scale of −3 to +3, with 0 indicating no change, the mean for all outcome items in the sample was 1.42, reflecting mild-to-moderate improvements across the items. Half of respondents (*N* = 1573; 50.0%) reported a score of +2 or +3 on at least half of the items (9 items or more). Mean scores and SDs of each individual positive outcome item with descending mean values are presented in Table 2. In order, the top three items with the greatest mean values were ‘Changes in my understanding of why I feel the way I do’, followed by ‘Changes in mood or reduced depression’ and ‘Changes in my understanding of my condition or how I relate to it’. All 17 items had a value above zero, indicating that, on average, respondents experienced positive change across all items.

**Table 2.** 17 Self-treatment outcome items with mean scores and SDs.

	Mean	SD
Changes in my understanding of why I feel the way I do	2.05	1.08
Changes in mood or reduced depression	1.89	1.10
Changes in my understanding of my condition or how I relate to it	1.82	1.15
Change in overall symptoms of your psychiatric condition	1.75	1.11
Changes in productivity, motivation or confidence	1.72	1.17
Changes in my tolerance towards others	1.70	1.28
Changes in empathy, sociability and communication skills	1.70	1.19
Changes in ability to control negative thoughts/persistent worryings	1.68	1.27
Changes in self-identity	1.54	1.23
Changes in life priorities	1.52	1.18
Changes in feelings of frustration/anger	1.38	1.31
Changes in energy, alertness and/or focus	1.31	1.25
Changes in anxiety, including social anxiety	1.27	1.26
Changes in my use of alcohol/other drugs	0.97	1.33
Changes in sight, smell or hearing	0.71	1.16
Changes in concentration/memory	0.60	1.25
Changes in sleep	0.39	1.16

SD: standard deviation.

94.6% of respondents had a value of +2 or +3 on at least one outcome item, and were therefore presented questions on the time-to-onset and duration of most positive outcomes. For the majority of these respondents, most positive outcomes were noticed within 24 h (64.3%; *N* = 1890); for 9.9% (*N* = 291) of respondents, experiencing most positive outcomes took more than a week. The reported duration of most positive effects was at least 4 weeks for 1497 respondents (52.7%), with 499 (17.6%) reporting experiences lasting over 6 months (Table 3).

**Table 3.** Duration of majority of positive and negative outcomes.

Positive outcomes			
	*N*	Valid %	Cum %
>12 h	105	3.7	3.7
12–24 h	129	4.5	8.2
1–2 days	217	7.6	15.9
3–7 days	256	9.0	24.9
1 week	218	7.7	32.6
2 weeks	270	9.5	42.1
3 weeks	147	5.2	47.3
4 weeks	191	6.7	54.0
1–2 months	454	16.0	70.0
3–4 months	263	9.3	79.3
5–6 months	90	3.2	82.4
>6 months	499	17.6	100.0
Total	2839	100.0	
Negative outcomes			
	*N*	Valid %	Cum %
>12 h	160	23.6	23.6
12–24 h	90	13.3	36.9
1–2 days	81	11.9	48.8
3–7 days	39	5.8	54.6
1 week	43	6.3	60.9
2 weeks	42	6.2	67.1
3 weeks	18	2.7	69.8
4 weeks	22	3.2	73.0
1–2 months	49	7.2	80.2
3–4 months	32	4.7	85.0
5–6 months	14	2.1	87.0
>6 months	88	13.0	100.0
Total	678	100.0	

Duration of positive outcomes was asked from those respondents only with a score of +2 or +3 on at least one self-treatment outcome item (*N* = 2975). Duration of negative outcomes was asked from those respondents only who indicated experiencing any negative outcomes (*N* = 705).

#### 10-Item negative outcome scale

**Figure 2.**
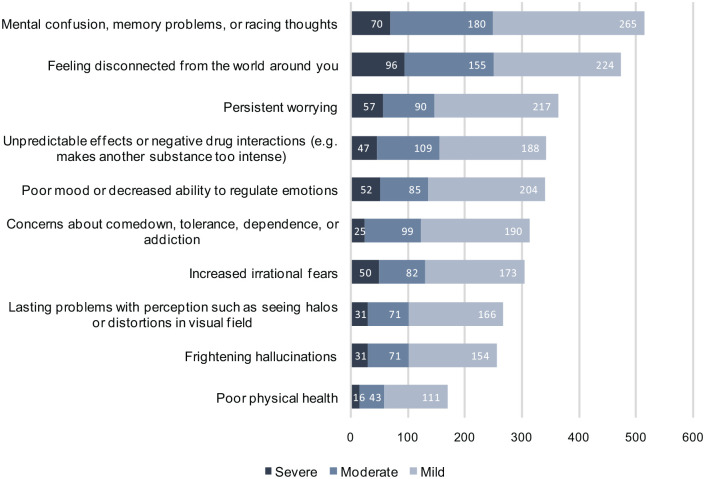
Frequencies of negative outcomes.

There were 705 respondents (22.5% out of 3136 responses on this question) who indicated experiencing at least one negative consequence from their experience. The median number of negative outcomes (of any severity) among these respondents was 5.0 (interquartile range = 3.0–7.0). Most commonly reported outcomes included ‘Mental confusion, memory problems, or racing thoughts’ and ‘Feeling disconnected from the world around you’ (Figure 2).

Most reported noticing at least one of the negative outcomes within 24 h (59.7%; *N* = 410); but for one-fifth of respondents who experienced at least one negative outcome the effect was noticed more than 1 week later (20.1%; *N* = 138). Duration of most negative outcomes was 7 days or less for 54.6% of respondents (*N* = 370) but lasted a month or more for 27.0% (*N* = 183; Table 3).

Of those reporting any negative outcomes, 4.2% (*N* = 28; 0.9% of the total sample) reported seeking emergency medical treatment following self-treatment with LSD or psilocybin mushrooms; 3.8% (*N* = 25) respondents once and 0.5% (*N* = 3) more than once.

### Treatment outcomes: Predictors

#### 17-Item self-treatment outcome scale

The model predicting scores on the 17-item outcome scale were fitted based on 3145 cases with valid responses on the outcome variable. All assumptions of multiple linear regression (normality, homoscedasticity and absence of multicollinearity) were met.

Results of the regression are presented in Table 4. The overall model was statistically significant, but only explained 6% of the variance in the outcome variable (*R*^2^ = 0.06, *F*[21, 3004] = 9.36, *p* < 0.001). After accounting for all other factors in the model, the results suggest self-treatment with LSD was associated with lower outcome scores compared to psilocybin mushrooms. Having sought advice/information before self-treatment was associated with higher outcome scores. The omnibus test (testing the overall significance of a categorical variable in the model) showed a significant effect of intensity of experience on outcome scores, *X*^2^(4) = 79.821, *p* < 0.001; lower intensities of experience were associated with lower outcome scores compared to the reference category ‘High intensity of experience’. Specifically, compared to intense psychedelic experience, ‘No experience or effects at all’ was associated with lowest outcome scores, while no notable difference was observed between effects of the three middle categories of intensity. The Omnibus test also revealed a significant effect of condition treated on outcome scores, *X*^2^(12) = 48.221, *p* < 0.001; specifically, treating post-traumatic stress disorder (PTSD) was associated with higher outcome scores compared to the reference category depression. Again, after accounting for all other factors in the model, no associations were observed between outcome scores and age or gender.

**Table 4.** Results of OLS regression predicting 17-item outcome scale scores.

	Unst. Coeff.		Std. Coeff.			99.8% Cl for *B*	
	*B*	SE	β	*t*	Sig.	Lower	Upper
Intercept	1.355	0.063		21.583 *	<0.001	1.161	1.549
Age	0.001	0.002	0.016	0.874	0.382	−0.004	0.007
Gender							
Female	−0.016	0.033	−0.009	−0.492	0.623	−0.120	0.087
Non-binary or other	0.036	0.074	0.009	0.480	0.631	−0.194	0.266
Male	Ref						
Substance							
LSD	−0.099	0.029	−0.062	−3.414 *	0.001	−0.189	−0.009
Psilocybin mushrooms	Ref						
Seeking advice							
Yes	0.221	0.035	0.112	6.252 *	<0.001	0.112	0.331
No	Ref						
Intensity of experience							
No experience or effects at all	−0.956	0.177	−0.096	−5.392 *	<0.001	−1.505	−0.408
No psychedelic experience, but other acute effects	−0.237	0.083	−0.052	−2.855	0.004	−0.493	0.020
Mild psychedelic experience	−0.200	0.047	−0.081	−4.284 *	<0.001	−0.344	−0.056
Moderate psychedelic experience	−0.217	0.031	−0.133	−7.103 *	<0.001	−0.312	−0.123
Intense psychedelic experience	Ref						
Primary condition treated							
Anxiety	0.052	0.038	0.027	1.377	0.169	−0.065	0.170
OCD	−0.171	0.172	−0.018	−0.994	0.320	−0.703	0.361
Bipolar	0.053	0.101	0.009	0.525	0.599	−0.260	0.367
PTSD	0.316	0.076	0.076	4.151 *	<0.001	0.081	0.552
Psychosis	0.007	0.192	0.001	0.038	0.970	−0.588	0.602
Alcohol or other SUD	0.007	0.069	0.002	0.108	0.914	−0.206	0.220
Anorexia/bulimia	−0.471	0.173	−0.049	−2.718	0.007	−1.007	0.065
Distress associated with a medical condition	−0.132	0.119	−0.020	−1.111	0.267	−0.499	0.235
Bereavement	−0.270	0.102	−0.048	−2.642	0.008	−0.586	0.046
Trauma	0.022	0.065	0.006	0.335	0.737	−0.180	0.224
Relationship problem	−0.073	0.051	−0.027	−1.422	0.155	−0.232	0.086
Other	0.126	0.049	0.048	2.560	0.011	−0.026	0.277
Depression	Ref						

Dependent variable: 17-item Outcome Scale. Model based on 3026 complete cases.

Coeff.: coefficient; LSD: lysergic acid diethylamide; OCD: obsessive compulsive disorder; OLS: ordinary least squares; PTSD: post-traumatic stress disorder; Ref: reference category; SE: standard error; Std.: standardised; SUD: substance use disorder; Unst.: unstandardized.

* *p* < 0.002.

To confirm the results of the regression were not affected by missing values, a sensitivity analysis was performed by conducting the same regression analysis among those respondents only with less than 5 missing values or N/As across the outcome items (*N* = 3041; regression model based on *N* = 2935 with 106 cases dropped due to missingness on predictors). Results of this analysis were comparable to the original regression, with the same predictor variables identified; see Supplemental Table S3.

#### Duration of positive outcomes

The model predicting duration of positive outcomes was fitted based on 2839 cases with valid responses on the outcome variable. Proportional odds assumption of ordinal regression was violated (*p* = 0.002); therefore, multinomial logistic regression was conducted, with the category of lowest duration (‘up to 7 days’) as the reference category.

Results of the regression model are presented in Table 5. After accounting for all other factors in the model, omnibus tests showed intensity of experience was the only variable predicting duration of positive outcomes, *X*^2^(8) = 292.346, *p* < 0.001. Specifically, lower intensities were associated with shorter durations. Compared to the reference category ‘High intensity of experience’, significant effects were found for ‘Mild psychedelic experience’ and ‘No psychedelic experience but other acute effects’ for both the comparisons between short and medium duration, and short and high duration of outcomes; and additionally, for ‘Moderate psychedelic experience’ for the comparison between short and high duration of outcomes. ‘No experience or effects at all’ did not reach significance on our adjusted *p* value possibly due to very small sample size in this group, with coefficients indicating effects to the same direction. The predicted probability of each level of experience intensity at each level of outcome duration, after accounting for other covariates in the model, is visualised in Supplemental Figure S1. No association was observed between duration of positive outcomes and age, gender, substance used, seeking advice, nor condition/problem treated.

**Table 5.** Results of multinomial logistic regression predicting duration of positive outcomes (outcome reference group ‘up to 7 days’).

	Exp (*B*)	Wald	Sig.	99.8% CI for Exp (*B*)		Exp (*B*)	Wald	Sig.	99.8% CI for Exp (*B*)	
				Lower	Upper				Lower	Upper
Intercept		10.558 *	0.001				30.650 *	<0.001		
Age	0.999	0.020	0.889	0.979	1.020	0.994	0.974	0.324	0.975	1.013
Gender										
Female	0.779	3.735	0.053	0.523	1.161	0.879	1.169	0.280	0.607	1.272
Non-binary or other	1.116	0.153	0.696	0.469	2.653	0.795	0.688	0.407	0.338	1.869
Male	Ref					Ref				
Substance										
LSD	0.790	4.276	0.039	0.555	1.124	0.762	6.432	0.011	0.547	1.061
Psilocybin mushrooms	Ref					Ref				
Seeking advice										
Yes	1.087	0.382	0.537	0.717	1.648	1.362	5.768	0.016	0.915	2.027
No	Ref					Ref				
Intensity of experience										
No experience or effects at all	0.087	5.149	0.023	0.003	2.421	0.178	7.288	0.007	0.025	1.284
No psychedelic experience, but other acute effects	0.132	38.711 *	<0.001	0.048	0.361	0.061	70.413 *	<0.001	0.022	0.171
Mild psychedelic experience	0.210	81.165 *	<0.001	0.123	0.359	0.113	171.096 *	<0.001	0.067	0.189
Moderate psychedelic experience	0.796	3.590	0.058	0.548	1.155	0.471	44.324 *	<0.001	0.332	0.668
Intense psychedelic experience	Ref					Ref				
Primary condition treated										
Anxiety	1.008	0.003	0.958	0.643	1.578	1.154	1.074	0.300	0.753	1.769
OCD	1.208	0.056	0.813	0.103	14.214	2.470	1.656	0.198	0.281	21.683
Bipolar	0.966	0.008	0.931	0.278	3.353	1.295	0.489	0.484	0.413	4.059
PTSD	1.012	0.002	0.968	0.404	2.536	1.330	1.061	0.303	0.565	3.130
Psychosis	0.963	0.002	0.961	0.087	10.715	1.185	0.054	0.817	0.123	11.391
Alcohol or other SUD	0.772	0.947	0.330	0.340	1.755	0.827	0.566	0.452	0.378	1.807
Anorexia/bulimia	0.265	2.597	0.107	0.021	3.381	1.010	0.000	0.985	0.193	5.284
Distress associated with a medical condition	0.890	0.064	0.801	0.215	3.695	1.053	0.014	0.904	0.278	3.987
Bereavement	1.081	0.028	0.867	0.255	4.588	1.968	2.595	0.107	0.537	7.209
Trauma	1.328	1.133	0.287	0.582	3.030	1.512	2.662	0.103	0.691	3.305
Relationship problem	0.945	0.080	0.777	0.510	1.750	1.063	0.105	0.746	0.594	1.904
Other	0.621	5.431	0.020	0.330	1.168	1.292	2.145	0.143	0.753	2.217
Depression	Ref					Ref				

Dependent variable: duration of positive outcomes (up to 7 days/1–4 weeks/1 month or more). Model based on 2761 complete cases.

LSD: lysergic acid diethylamide; OCD: obsessive compulsive disorder; PTSD: post-traumatic stress disorder; Ref: reference category; SUD: substance use disorder.

* *p* < 0.002.

#### 10-Item Negative Outcome Scale

The negative binomial regression model predicting number of negative outcomes was fitted based on 3136 cases with valid responses on the outcome variable. The results of the model are presented in Table 6. After accounting for all other factors in the model, older age was associated with a lower number of negative outcomes. Furthermore, LSD was associated with a higher number of negative outcomes compared to psilocybin mushrooms. Finally, the omnibus test showed intensity of experience was associated with number of negative outcomes, *X*^2^(4) = 35.646, *p* < 0.001. Specifically, ‘no psychedelic experience but other acute effects’ and ‘moderate psychedelic experience’ were associated with lower number of negative outcomes, compared to highest intensity of experience. No association was observed between number of negative outcomes and gender, seeking advice, nor condition/problem treated.

**Table 6.** Results of negative binomial regression predicting number of negative outcomes.

	Exp(*B*)	Wald	Sig.	99.8% CI for Exp(*B*)	
				Lower	Upper
Intercept	3.111	79.324 *	<0.001	2.098	4.612
Age	0.954	141.795 *	<0.001	0.942	0.965
Gender					
Female	1.119	3.208	0.073	0.922	1.357
Non-binary or other	1.206	1.932	0.165	0.796	1.827
Male	Ref				
Substance					
LSD	1.586	67.333 *	<0.001	1.333	1.887
Psilocybin mushrooms	Ref				
Seeking advice					
Yes	0.865	4.878	0.027	0.707	1.059
No	Ref				
Intensity of experience					
No experience or effects at all	0.764	0.519	0.471	0.241	2.423
No psychedelic experience, but other acute effects	0.417	16.935 *	<0.001	0.216	0.804
Mild psychedelic experience	0.840	3.781	0.052	0.636	1.108
Moderate psychedelic experience	0.769	20.785 *	<0.001	0.644	0.919
Intense psychedelic experience	Ref				
Primary condition treated					
Anxiety	0.977	0.109	0.742	0.784	1.216
OCD	1.361	1.058	0.304	0.539	3.437
Bipolar	1.288	2.109	0.146	0.751	2.209
PTSD	0.863	0.888	0.346	0.532	1.400
Psychosis	1.072	0.047	0.828	0.400	2.875
Alcohol or other SUD	0.730	5.029	0.025	0.473	1.126
Anorexia/bulimia	0.894	0.138	0.710	0.351	2.276
Distress associated with a medical condition	1.036	0.025	0.874	0.520	2.066
Bereavement	0.899	0.279	0.597	0.483	1.674
Trauma	1.210	2.650	0.104	0.842	1.739
Relationship problem	0.840	3.222	0.073	0.622	1.134
Other	0.883	1.787	0.181	0.661	1.178
Depression	Ref				

Dependent variable: Number of negative outcomes. Model based on 3046 complete cases.

CI: confidence interval; LSD: lysergic acid diethylamide; OCD: obsessive compulsive disorder; PTSD: post-traumatic stress disorder; Ref: reference category; SUD: substance use disorder.

* *p* < 0.002.

#### Duration of negative outcomes

The model predicting duration of negative outcomes was fitted based on 678 cases with valid responses on the outcome variable. Proportional odds assumption of the ordinal regression was met (*p* = 0.849); therefore, results of this analysis are reported: the estimates of the threshold/cut points are presented in Table 7 along with the results of the ordinal regression model. Unlike the results presented in Tables 4 to 6, the only significant predictor of duration of negative outcomes was age; specifically, older age was associated with lower duration of negative outcomes. No association was observed between duration of negative outcomes and gender, substance used, seeking advice, intensity of experience, nor condition/problem treated.

**Table 7.** Results of ordinal regression predicting duration of negative outcomes.

		Exp (*B*)	Wald	Sig.	99.8% CI for Exp (*B*)	
					Lower	Upper
Threshold	Duration of negative outcomes = up to 7 days	0.213	13.091 *	<0.001	0.056	0.787
	Duration of negative outcomes = 1–4 weeks	0.506	2.571	0.109	0.134	1.857
Age		0.941	19.145 *	<0.001	0.900	0.981
Gender						
Female		0.990	0.003	0.957	0.556	1.745
Non-binary or other		0.445	3.843	0.050	0.108	1.484
Male		Ref				
Substance						
LSD		1.022	0.015	0.902	0.590	1.783
Psilocybin mushrooms		Ref				
Seeking advice						
Yes		0.814	1.055	0.304	0.440	1.522
No		Ref				
Intensity of experience						
No experience or effects at all		0.850	0.027	0.869	0.020	20.438
No psychedelic experience, but other acute effects		0.291	2.453	0.117	0.009	2.275
Mild psychedelic experience		0.534	4.896	0.027	0.213	1.248
Moderate psychedelic experience		0.801	1.661	0.197	0.469	1.360
Intense psychedelic experience		Ref				
Primary condition treated						
Anxiety		1.306	1.632	0.201	0.683	2.490
OCD		3.630	1.835	0.176	0.165	145.937
Bipolar		1.449	0.689	0.406	0.347	5.932
PTSD		0.897	0.049	0.824	0.167	3.842
Psychosis		1.766	0.668	0.414	0.188	19.659
Alcohol or other SUD		0.883	0.084	0.772	0.214	3.216
Anorexia/bulimia		0.531	0.286	0.593	0.001	15.715
Distress associated with a medical condition		1.237	0.174	0.677	0.233	6.137
Bereavement		2.460	1.422	0.233	0.199	30.814
Trauma		1.084	0.059	0.808	0.372	2.991
Relationship problem		0.606	2.796	0.094	0.230	1.495
Other		0.640	2.381	0.123	0.251	1.528
Depression		Ref				

Dependent variable: duration of negative outcomes. Model based on 663 complete cases.

CI: confidence interval; LSD: lysergic acid diethylamide; OCD: obsessive compulsive disorder; PTSD: post-traumatic stress disorder; Ref: reference category; SUD: substance use disorder.

* *p* < 0.002.

## Discussion

This present study investigated patterns and outcomes of psychedelic self-treatment using data from a large international survey of 3364 people reporting on their self-treatment experiences with LSD or psilocybin mushrooms. The most commonly such treated conditions were depression and anxiety, accounting for 60% of the sample. Reported outcomes were generally positive, average outcome scores reflecting mild-to-moderate improvements across items, which lasted beyond 3 weeks for the majority of respondents. 22.5% of participants reported experiencing some negative consequences from their experience but these were rarely severe or long lasting. A number of significant outcome predictors were identified, namely intensity of psychedelic experience, substance used, seeking advice beforehand, condition treated and age. The time scales for reported improvements closely mirror those seen in clinical trials.

The top-rated positive outcome items were ‘Changes in understanding of why I feel the way I do’ and ’Changes in my understanding of my condition and how I relate to it’. Increased insights into oneself and one’s condition are one of the most commonly reported psychedelic-induced therapeutic processes and outcomes described both by participants in clinical trials and users in the community (Breeksema et al., 2020; Soares et al., 2022). Increased insight obtained during or immediately after a psychedelic experience has been found to be associated with longer-term increases in well-being (Davis et al., 2021b; Peill et al., 2022), mediating the relationship between acute emotional breakthrough and subsequent well-being (Peill et al., 2022). Although only a proportion of patients undergoing psychedelic therapy achieve full remission from their psychiatric symptoms, it has been suggested that increased psychological insight obtained from the treatment can improve the ability to manage one’s condition and respond to subsequent stressors more adaptively, contributing to sustained outcomes and supporting long-term mental health (Peill et al., 2022). Overall, respondents in our survey reported improvements across a wide range of outcomes, with all item averages above zero indicating a positive change. The findings are consistent with previous literature highlighting psychedelics’ potential to induce holistic and transdiagnostic therapeutic effects (Kočárová et al., 2021).

Reported negative outcomes were mostly related to emotional states as well as some cognitive functions and appeared largely similar to those observed from recreational use (Barrett et al., 2016; Bienemann et al., 2020; Carbonaro et al., 2016; Gashi et al., 2021; Soares et al., 2022). Lasting problems or distortions in perception were reported by 268 respondents corresponding to 8.5% of all self-treaters; however, only 11.6% of those regarded them as severe. These results largely reflect previous research showing such phenomena are not uncommon, but are rarely cited as disabling or severely distressing and therefore rarely meet the criteria for hallucinogen persisting perception disorder (HPPD) recognised in DSM-5 (Carhart-Harris and Nutt, 2010; Muller et al., 2022). Such experiences may represent an enduring effect of psychedelics on visual processing, or may represent an increased awareness of the normal breadth of visual experience (Anthony et al., 2020).

Experienced negative effects were usually short lasting, but for a quarter of those reporting negative outcomes, these lasted for over 4 weeks. Based on available evidence, the risks of persisting negative effects appear lower in clinical settings. A review of psychedelic trials conducted at Johns Hopkins, including a total of 250 individuals (both healthy and clinical populations) treated in more than 380 psychedelic sessions up until 2016, found only three participants had reported transient negative psychological effects, all of which eventually resolved (Carbonaro et al., 2016). The first treatment-related serious adverse effects were observed only recently in two clinical trials, which reported suicidality, self-harm and psychiatric symptoms in 2–5% of patients receiving an active dose of psilocybin or LSD (Goodwin et al., 2022a; Holze et al., 2023). No cases of HPPD, sustained psychosis, or sustained cognitive impairment have been reported in modern clinical research (Bender and Hellerstein, 2022; Rucker et al., 2022). Several cases of re-emergence of traumatic memories during or shortly after treatment have been observed (Bender and Hellerstein, 2022; Johnson et al., 2008; Watts et al., 2017), but in most studies, any resulting distress was reported as resolved during subsequent integration or counselling sessions (Johnson et al., 2017; Studerus et al., 2011; Timmermann et al., 2020). In self-treatment contexts, a lack of professional interpersonal support may increase the risk of longer-term difficulties from similar experiences (Glynos et al., 2022). Of note, we highlight that recording individual sub-acute adverse effects in psychedelic clinical trials may sometimes be considered inadequate, as assessment of longer-term outcomes is generally focussed on mean changes in symptom scale ratings, and may further be affected by experimenter biases and social desirability bias (Breeksema et al., 2022; Muthukumaraswamy et al., 2021; Muthukumaraswamy et al., 2022). Information obtained from our survey was also limited, as durations of outcomes were not rated individually for each item, and the provided list of outcomes was non-exhaustive and lacked an option for an open-ended answer. Despite the relatively low prevalence of severe or persisting adverse effects observed in response to psychedelics, there is an ethical duty for increased research on the predictors, nature, and management of these both in and outside clinical settings, with increasing numbers of people likely to undergo psychedelic experiences.

A large majority of respondents had sought advice or information before using psychedelics for self-treatment. Similarly to previous research on psychedelic use (Glynos et al., 2022; Shaw et al., 2022), most had obtained advice from online sources, friends or family, while advice from a therapist or doctor was very rarely reported. Having sought advice predicted higher outcome scores and showed a trend towards longer duration of positive outcomes and lower number of negative outcomes. Although these results together indicate informal sources of advice are seemingly useful in most cases, reliance on these also suggests limited availability of reliable and evidence-based harm reduction resources from authorities. Education of therapists and other health and social care workers, alongside efforts to reduce stigma associated with psychedelics and other drugs, could be particularly important to help ensure provision of personal support for people using psychedelics (Glynos et al., 2022; Gorman et al., 2021; Pilecki et al., 2021), the importance of which we highlighted previously (Breeksema et al., 2022; Glynos et al., 2022; Johnson et al., 2008; Murphy et al., 2022).

The highest intensity of experience was associated with higher outcome scores compared to other intensity levels. This was also related to longer-lasting positive outcomes, with coefficients at each level indicating a linear relationship between intensity and duration. Most modern psychedelic trials use moderate–high doses, aimed to induce ‘peak’ or ‘mystical-type’ experiences and/or emotional breakthrough, that have been found to predict symptomatic improvement (Davis et al., 2020; Goodwin et al., 2022b; Ko et al., 2022; Yaden and Griffiths, 2020). Superior efficacy of high doses was also supported by a recent systematic review that identified higher intensity of experience as the main predictor of favourable outcomes from psychedelic treatments (Romeo et al., 2021); and by a large multicentre trial on psilocybin for depression where a high 25-mg dose demonstrated better outcomes compared to a low–moderate 10-mg dose (Goodwin et al., 2022a). However, the highest intensity of experience was also related to more negative outcomes in our study, consistent with previous research showing increasing dosages correlate both with increasing positive and negative acute and sub-acute effects (Griffiths et al., 2011; Holze et al., 2022; Studerus et al., 2011; Studerus et al., 2012). In both the present study and previous clinical trials, favourable outcomes have also been observed with moderate doses or less intense experiences (Gasser et al., 2015; Grob et al., 2011; Johnson et al., 2014; Moreno et al., 2006; Nielson et al., 2018; Roseman et al., 2018), which may provide a suitable alternative for those not willing or safely able to experience the full effects of high-dose psychedelics or where extended clinical supervision is not available. In two clinical trials on psilocybin-assisted therapy for depression, an initial moderate dose was administered first to familiarise participants with the drug effects before a high-dose session a few weeks afterwards (Carhart-Harris et al., 2016; Davis et al., 2021a). These studies reported larger treatment effects to trials using a single high dose, but more research would be needed to confirm the comparative efficacy and importantly, comparative safety and cost-effectiveness of different dosing regimens (Leger and Unterwald, 2022).

Treating with psilocybin mushrooms was associated with higher outcome scores compared to LSD, although with a very small effect. Psilocybin mushrooms were also associated with fewer negative outcomes, reflecting previous findings on recreational use (Kopra et al., 2022b; Leonard et al., 2018). Modern clinical trials on psychedelics have primarily been conducted with psilocybin in contrast to pre-prohibition era trials mostly using LSD; this change was presumably driven by the lower level of stigma associated with psilocybin and its lower duration of action, but now seems to be supported by preliminary evidence of the drugs’ comparative safety profile. Evidence regarding potential differences in therapeutic efficacy remains, however, inconclusive. In a previous survey study by Carhart-Harris and Nutt (2010), psychedelic users rated LSD’s therapeutic potential and perceived benefits on well-being and physical/mental health marginally higher than psilocybin’s. Two recent systematic reviews on outcome predictors of psychedelic treatments found no effect of the specific psychedelic used (Leger and Unterwald, 2022; Romeo et al., 2021); although these analyses were highly limited due to small number of studies especially with LSD, as well as high heterogeneity between sample characteristics and methodologies. The first modern experimental study directly comparing the subjective effects of LSD and psilocybin in healthy individuals, adapting a placebo-controlled cross-over design, likewise found largely comparable effects between the substances apart from the longer duration of LSD (Holze et al., 2022). Regardless, the presence of more subtle (yet clinically relevant) differences in the experience not captured by the administered measures cannot be ruled out, and may even be expected given for instance the dopaminergic action observed with LSD but not with other classical psychedelics (which, of note, could also account for the higher frequency of adverse effects observed from LSD; Rickli et al., 2016). In the present survey, individuals who had self-treated with both LSD and psilocybin mushrooms showed an equal split regarding which drug they indicated preferring more. It is plausible that some specific properties of each substance suit people differently – and higher variability between outcomes related to each substance could be observed within individual subjects, and/or between specific outcomes, than between group averages of total outcomes. An investigation is currently underway comparing intensity and occurrence of specific positive and negative self-treatment outcomes between LSD, psilocybin mushrooms and MDMA, using GDS2020 data.

Psychedelics have demonstrated transdiagnostic potential since early research (Butler et al., 2020; Kočárová et al., 2021; Rucker et al., 2016; Weston et al., 2020), and our study likewise observed favourable treatment outcomes across various indications treated. These included previously little investigated conditions such as PTSD, bipolar disorder, eating disorders and psychosis; as well as various problems or worries not representing a diagnostic entity in themselves. Respondents treating PTSD demonstrated highest outcome scores on the 17-item scale, with significantly higher scores to the reference category of depression. The potential of classical psychedelics for the treatment of PTSD has been the topic of several recent review articles (Bird et al., 2021; Elsouri et al., 2022; Henner et al., 2022; Khan et al., 2022; Krediet et al., 2020) and our results bring further validation for the first clinical trials on psilocybin-assisted therapy for PTSD currently underway (NCT05243329, NCT05312151, NCT05554094). However, we highlight that the apparent superiority of outcomes in PTSD in our study could be at least partially attributed to the wide range of comorbidity and impairment associated with this condition (Karatzias et al., 2019; Zayfert et al., 2002), allowing more substantial improvement on a higher number of outcome items. Besides the unmeasured variability in the range and severity of baseline symptoms, comparisons between conditions are further limited by small *N*s in many rarer conditions, resulting in low power to detect potential associations. In light of the rapidly expanding range of conditions, psychedelic treatments are being trialled for, it is nevertheless reassuring that no indication showed a trend towards increased negative consequences, providing preliminary evidence of psychedelics safety in these conditions.

Younger age was related to both higher number and longer duration of negative outcomes. Previously, younger age has been found associated with increased unpleasant or challenging experiences with psychedelics (Carbonaro et al., 2016; Studerus et al., 2012) as well as higher rate of incidents leading to emergency medical treatment seeking following psychedelic use (Kopra et al., 2022a; Kopra et al., 2022b). A recent survey study found a correlation between younger age and problematic psychedelic use, which became non-significant after adjusting for variables related to patterns of use (of which higher frequency of use and social/recreational intentions were linked to problematic use; St Arnaud and Sharpe, 2022). The findings may indicate younger age to be associated with ‘riskier’ patterns of psychedelic use, that at least partially mediate associations with increased adverse effects. No correlation was observed between age and scores on the 17-item treatment outcome scale, suggesting equal overall treatment benefits across age groups.

This study has several important limitations. First, self-selective sampling may disproportionately reach and attract certain characteristics. Our sample was predominantly white, young and educated, and although this appears to partly reflect the common demographic profile of psychedelic users (Yockey et al., 2020), GDS may also disproportionately reach these populations (Barratt et al., 2017). Low representation of ethnic minorities and socioeconomically disadvantaged individuals is a significant problem in psychedelic research, especially considering these populations are greatly affected by the mental conditions that any future psychedelic therapy would be used to treat (Ortiz et al., 2022; Rea and Wallace, 2021; Williams et al., 2021); and recent evidence does suggest the psychedelic user population is becoming increasingly ethnically diverse (Davis et al., 2022). Furthermore, those reached by the survey advertising and who choose to volunteer may have specific interests and knowledge about the topics of the survey (Eysenbach and Wyatt, 2002); and for instance be better educated about optimal practices of psychedelic use, contributing to more favourable outcomes.

Retrospective self-reports are vulnerable to recall biases, and personal opinions and attempts to influence survey results may also affect responses. We note that as our survey covers the use of a wide range of substances and is advertised on platforms not directly related to psychedelic use, influence of both response and selection biases may be attenuated in our survey, compared to surveys exclusively focussed on psychedelic use which observe highly favourable attitudes towards psychedelics among respondents (Haijen et al., 2018) .

Regardless, even if a representative sample of psychedelic self-treaters was obtained, these individuals likely hold positive expectations about the treatment; while resuming or discontinuing psychedelic use based on experiences before the survey reporting period induces an effect comparable to attrition or survivorship bias. Expectancy effects and volunteer biases are also issues in clinical trials across fields, but are particularly pronounced in psychedelic research due to the prevailing ‘hype’ around these treatments, and the strong effect of expectations observed particularly in responses to psychedelics (Aday et al., 2022; Butler et al., 2022; Muthukumaraswamy et al., 2021). Nonetheless, if some experienced users had observed the majority of psychedelics’ benefits during use before the survey reporting period, some of these effects may be attenuated in our study (Haijen et al., 2018).

The non-randomised, observational nature of the investigation also affects the assessment of the predictive value of some variables; as each respondent has, for instance, been able to choose the dose they consider most suitable for themselves. Crucially, respondents were asked to report on their experiences with the psychedelic they had found most useful in case they had self-treated with many. These effects could result both in more favourable outcomes overall, and more equal outcomes between substances and between intensities, than what might be observed in a randomised experiment. That generally favourable outcomes were observed across predictors does not necessarily indicate those predictors matter little, but rather that different things may work for different individuals.

The administered outcome measurements also have limitations. Although our 17-item outcome scale demonstrated high internal consistency, the assumptions of Cronbach’s alpha are often violated and other tests would ideally be run to for instance examine the factor loadings of each item (Agbo, 2010; Tavakol and Dennick, 2011). Furthermore, baseline levels of morbidity across items remain unknown; as discussed previously, this may particularly bias outcome comparisons between conditions, favouring conditions with higher range and severity of impairment. Furthermore, no validated symptom scales were used, limiting comparison of results to existing research. However, the use of such scales would have been impractical given the wide range of conditions investigated; and we also highlight the relevance of measuring broader aspects of wellbeing and functioning beyond symptoms attributed to a specific condition, which has been emphasised in modern mental health research (Huppert and So, 2013). Regarding duration of outcomes, given our survey enquired about past-year experiences, the prevalence of longer-lasting effects may have been underestimated in cases where respondents have experienced sustained outcomes at the time of reporting, but with not enough time having passed since the experience to report longer durations. We also did not record duration of outcomes for each item separately.

The variance explained by our regression models was small. Responses to psychedelics have generally been found difficult to predict (Aday et al., 2021); yet, there were several unmeasured variables of interest with potential relationship to outcomes such as the presence and quality of interpersonal support before, during and after the experience (Breeksema et al., 2020; Sepeda et al., 2020); expectations (Aday et al., 2022); as well as mental state and certain personality characteristics (Studerus et al., 2012). These and other unknown variables may have also acted as hidden confounders in our regression analyses. Finally, we cannot confirm the purity of substances used, and did not enquire about the dose used for self-treatment nor about potential other drugs or medications used concomitantly.

To summarise, this study sheds light on individual self-administered therapeutic use of LSD and psilocybin mushrooms, a practice which appears to be increasing but is little researched. We utilised a large, global sample, representing a diverse range of target conditions and problems. Self-treatment outcomes were generally favourable, with benefits observed across a wide range of outcomes and indications. Intensity of experience was among the strongest predictors of positive outcomes, being associated with not only stronger but also longer-lasting treatment effects. Persisting negative outcomes were relatively uncommon but appear more frequent than in clinical settings. Our findings can contribute to harm reduction efforts, as well as inform experimental research about potential risks, benefits and underlying therapeutic mechanisms of psychedelics. Future research can be improved with inclusion of additional predictor variables of interest, and utilisation of prospective designs including measurement of baseline symptoms.

## Supplemental Material

sj-docx-1-jop-10.1177_02698811231158245 – Supplemental material for Investigation of self-treatment with lysergic acid diethylamide and psilocybin mushrooms: Findings from the Global Drug Survey 2020 Click here for additional data file. Supplemental material, sj-docx-1-jop-10.1177_02698811231158245 for Investigation of self-treatment with lysergic acid diethylamide and psilocybin mushrooms: Findings from the Global Drug Survey 2020 by Emma I Kopra, Jason A Ferris, Adam R Winstock, Kim PC Kuypers, Allan H Young and James J Rucker in Journal of Psychopharmacology

## Notes

1 The more colloquial term ‘magic mushrooms’ was used in the survey, but is exchanged for ‘psilocybin mushrooms’ elsewhere in this manuscript.

## References

[bibr1-02698811231158245] Aday JS Davis AK Mitzkovitz CM , et al. (2021) Predicting reactions to psychedelic drugs: A systematic review of states and traits related to acute drug effects. ACS Pharmacol Transl Sci 4: 424–435. 33860172 10.1021/acsptsci.1c00014 PMC8033773

[bibr2-02698811231158245] Aday JS Heifets BD Pratscher SD , et al. (2022) Great expectations: Recommendations for improving the methodological rigor of psychedelic clinical trials. Psychopharmacology 239: 1989–2010. 35359159 10.1007/s00213-022-06123-7 PMC10184717

[bibr3-02698811231158245] Agbo AA (2010) Cronbach’s alpha: Review of limitations and associated recommendations. J Psychol Afr 20: 233–239.

[bibr4-02698811231158245] Agin-Liebes G Haas TF Lancelotta R , et al. (2021) Naturalistic use of mescaline is associated with self-reported psychiatric improvements and enduring positive life changes. ACS Pharmacol Transl Sci 4: 543–552. 33860184 10.1021/acsptsci.1c00018 PMC8033766

[bibr5-02698811231158245] Andersen KA Carhart-Harris R Nutt DJ , et al. (2021) Therapeutic effects of classic serotonergic psychedelics: A systematic review of modern-era clinical studies. Acta Psychiatr Scand 143: 101–118. 33125716 10.1111/acps.13249

[bibr6-02698811231158245] Anthony J Winstock A Ferris JA , et al. (2020) Improved colour blindness symptoms associated with recreational psychedelic use: Results from the Global Drug Survey 2017. Drug Sci Policy Law 6: 2050324520942345.

[bibr7-02698811231158245] Australian Institute of Health and Welfare (2020) National Drug Strategy Household Survey 2019. Canberra: AIHW.

[bibr8-02698811231158245] Barratt MJ Ferris JA Zahnow R , et al. (2017) Moving on from representativeness: Testing the utility of the Global Drug Survey. Subst Abuse 11: 1178221817716391. 28924351 10.1177/1178221817716391 PMC5595253

[bibr9-02698811231158245] Barrett FS Bradstreet MP Leoutsakos JS , et al. (2016) The challenging experience questionnaire: Characterization of challenging experiences with psilocybin mushrooms. J Psychopharmacol 30: 1279–1295. 27856683 10.1177/0269881116678781 PMC5549781

[bibr10-02698811231158245] Bender D Hellerstein DJ (2022) Assessing the risk–benefit profile of classical psychedelics: A clinical review of second-wave psychedelic research. Psychopharmacology 239: 1907–1932. 35022823 10.1007/s00213-021-06049-6

[bibr11-02698811231158245] Bienemann B Ruschel NS Campos ML , et al. (2020) Self-reported negative outcomes of psilocybin users: A quantitative textual analysis. PLoS One 15: e0229067. 10.1371/journal.pone.0229067 PMC7034876 32084160

[bibr12-02698811231158245] Bird CI Modlin NL Rucker JJ (2021) Psilocybin and MDMA for the treatment of trauma-related psychopathology. Int Rev Psychiatry 33: 229–249. 34121583 10.1080/09540261.2021.1919062

[bibr13-02698811231158245] Breeksema JJ Kuin BW Kamphuis J , et al. (2022) Adverse events in clinical treatments with serotonergic psychedelics and MDMA: A mixed-methods systematic review. J Psychopharmacol 36: 1100–1117. 36017784 10.1177/02698811221116926 PMC9548934

[bibr14-02698811231158245] Breeksema JJ Niemeijer AR Krediet E , et al. (2020) Psychedelic treatments for psychiatric disorders: A systematic review and thematic synthesis of patient experiences in qualitative studies. CNS Drugs 34: 925–946. 32803732 10.1007/s40263-020-00748-y PMC7447679

[bibr15-02698811231158245] Butler M Jelen L Rucker J (2022) Expectancy in placebo-controlled trials of psychedelics: If so, so what? Psychopharmacology 239: 3047–3055. 36063208 10.1007/s00213-022-06221-6 PMC9481484

[bibr16-02698811231158245] Butler M Seynaeve M Bradley-Westguard A , et al. (2023) Views on using psychoactive substances to self-manage functional neurological disorder: Online patient survey results. J Neuropsychiatry Clin Neurosci 35: 77–85. 35578800 10.1176/appi.neuropsych.21080213

[bibr17-02698811231158245] Butler M Seynaeve M Nicholson TR , et al. (2020) Psychedelic treatment of functional neurological disorder: A systematic review. Ther Adv Psychopharmacol 10: 2045125320912125. 32435447 10.1177/2045125320912125 PMC7225815

[bibr18-02698811231158245] Carbonaro TM Bradstreet MP Barrett FS , et al. (2016) Survey study of challenging experiences after ingesting psilocybin mushrooms: Acute and enduring positive and negative consequences. J Psychopharmacol 30: 1268–1278. 27578767 10.1177/0269881116662634 PMC5551678

[bibr19-02698811231158245] Carhart-Harris R Bolstridge M Rucker J , et al. (2016) Psilocybin with psychological support for treatment-resistant depression: an open-label feasibility study. Lancet Psychiatry 3: 619–627. 27210031 10.1016/S2215-0366(16)30065-7

[bibr20-02698811231158245] Carhart-Harris RL Nutt DJ (2010) User perceptions of the benefits and harms of hallucinogenic drug use: A web-based questionnaire study. J Subst Use 15: 283–300.

[bibr21-02698811231158245] Carhart-Harris RL Roseman L Haijen E , et al. (2018) Psychedelics and the essential importance of context. J Psychopharmacol 32: 725–731. 29446697 10.1177/0269881118754710

[bibr22-02698811231158245] Carhart-Harris RL Wagner AC Agrawal M , et al. (2022) Can pragmatic research, real-world data and digital technologies aid the development of psychedelic medicine? J Psychopharmacol 36: 6–11. 33888025 10.1177/02698811211008567 PMC8801625

[bibr23-02698811231158245] Carod-Artal FJ (2015) Hallucinogenic drugs in pre-Columbian Mesoamerican cultures. Neurología 30: 42–49. 21893367 10.1016/j.nrl.2011.07.003

[bibr24-02698811231158245] Davis AK Arterberry BJ Xin Y , et al. (2022) Race, ethnic, and sex differences in prevalence of and trends in hallucinogen consumption among lifetime users in the United States between 2015 and 2019. Front Epidemiol 2: 3. 10.3389/fepid.2022.876706 PMC10910982 38455323

[bibr25-02698811231158245] Davis AK Barrett FS Griffiths RR (2020) Psychological flexibility mediates the relations between acute psychedelic effects and subjective decreases in depression and anxiety. J Contextual Behav Sci 15: 39–45. 32864325 10.1016/j.jcbs.2019.11.004 PMC7451132

[bibr26-02698811231158245] Davis AK Barrett FS May DG , et al. (2021a) Effects of psilocybin-assisted therapy on major depressive disorder a randomized clinical trial. JAMA Psychiatry 78: 481–489. 33146667 10.1001/jamapsychiatry.2020.3285 PMC7643046

[bibr27-02698811231158245] Davis AK Barrett FS So S , et al. (2021b) Development of the psychological insight questionnaire among a sample of people who have consumed psilocybin or LSD. J Psychopharmacol 35: 437–446. 33427007 10.1177/0269881120967878 PMC8056708

[bibr28-02698811231158245] Dos Santos RG Hallak JE Baker G , et al. (2021) Hallucinogenic/psychedelic 5HT2A receptor agonists as rapid antidepressant therapeutics: Evidence and mechanisms of action. J Psychopharmacol 35: 453–458. 33740877 10.1177/0269881120986422

[bibr29-02698811231158245] Elsouri KN Kalhori S Colunge D , et al. (2022) Psychoactive drugs in the management of post traumatic stress disorder: A promising New Horizon. Cureus 14: e25235. 10.7759/cureus.25235 PMC9214830 35747039

[bibr30-02698811231158245] Eysenbach G Wyatt J (2002) Using the Internet for surveys and health research. J Med Internet Res 4: e13. 10.2196/jmir.4.2.e13 PMC1761932 12554560

[bibr31-02698811231158245] Garcia-Romeu A Davis AK Erowid E , et al. (2020) Persisting reductions in cannabis, opioid, and stimulant misuse after naturalistic psychedelic use: An online survey. Front Psychiatry 10: 955. 32038317 10.3389/fpsyt.2019.00955 PMC6987443

[bibr32-02698811231158245] Garcia-Romeu A Davis AK Erowid F , et al. (2019) Cessation and reduction in alcohol consumption and misuse after psychedelic use. J Psychopharmacol 33: 1088–1101. 31084460 10.1177/0269881119845793

[bibr33-02698811231158245] Gashi L Sandberg S Pedersen W (2021) Making “bad trips” good: How users of psychedelics narratively transform challenging trips into valuable experiences. Int J Drug Policy 87: 102997. 33080454 10.1016/j.drugpo.2020.102997

[bibr34-02698811231158245] Gasser P Kirchner K Passie T (2015) LSD-assisted psychotherapy for anxiety associated with a life-threatening disease: A qualitative study of acute and sustained subjective effects. J Psychopharmacol 29: 57–68. 25389218 10.1177/0269881114555249

[bibr35-02698811231158245] Glynos NG Fields CW Barron J , et al. (2022) Naturalistic psychedelic use: A world apart from clinical care. J Psychoactive Drugs 11: 1–10. 10.1080/02791072.2022.2108356 35950817

[bibr36-02698811231158245] Goodwin GM Aaronson ST Alvarez O , et al. (2022a) Single-dose psilocybin for a treatment-resistant episode of major depression. N Engl J Med 387: 1637–1648. 36322843 10.1056/NEJMoa2206443

[bibr37-02698811231158245] Goodwin GM Feifel D Hellerstein DJ , et al. (2022b) Dose-dependent acute subjective psychedelic effects following COMP360 psilocybin across three clinical studies and its relationship to therapeutic response. [Poster]. In: 61st ACNP Annual Meeting, Phoenix, Arizona, 4–7 December 2022.

[bibr38-02698811231158245] Gorman I Nielson EM Molinar A , et al. (2021) Psychedelic harm reduction and integration: A transtheoretical model for clinical practice. Front Psychol 12: 645246. 33796055 10.3389/fpsyg.2021.645246 PMC8008322

[bibr39-02698811231158245] GOV.UK (2019) Crime Survey for England and Wales (CSEW) 2019. Available at: https://www.gov.uk/government/statistics/drug-misuse-findings-from-the-2018-to-2019-csew (accessed 6 January 2023).

[bibr40-02698811231158245] Griffiths RR Johnson MW Richards WA , et al. (2011) Psilocybin occasioned mystical-type experiences: Immediate and persisting dose-related effects. Psychopharmacology 218: 649–665. 21674151 10.1007/s00213-011-2358-5 PMC3308357

[bibr41-02698811231158245] Grob CS Danforth AL Chopra GS , et al. (2011) Pilot study of psilocybin treatment for anxiety in patients with advanced-stage cancer. Arch Gen Psychiatry 68: 71–78. 20819978 10.1001/archgenpsychiatry.2010.116

[bibr42-02698811231158245] Guerra-Doce E (2015) Psychoactive substances in prehistoric times: Examining the archaeological evidence. Time Mind 8: 91–112.

[bibr43-02698811231158245] Haijen EC Kaelen M Roseman L , et al. (2018) Predicting responses to psychedelics: A prospective study. Front Pharmacol 9: 897. 30450045 10.3389/fphar.2018.00897 PMC6225734

[bibr44-02698811231158245] Henner RL Keshavan MS Hill KP (2022) Review of potential psychedelic treatments for PTSD. J Neurol Sci 439: 120302. 35700643 10.1016/j.jns.2022.120302

[bibr45-02698811231158245] Hofmann A Heim R Brack A , et al. (1959) Psilocybin und psilocin, zwei psychotrope Wirkstoffe aus mexikanischen Rauschpilzen. Helv Chim Acta 42: 1557–1572.

[bibr46-02698811231158245] Hofmann A Ott J (1980) LSD, My Problem Child. New York: McGraw-Hill.

[bibr47-02698811231158245] Holze F Gasser P Müller F , et al. (2023) Lysergic acid diethylamide–assisted therapy in patients with anxiety with and without a life-threatening illness: A randomized, double-blind, placebo-controlled phase II study. Biol Psychiatry 93: 215–223. 36266118 10.1016/j.biopsych.2022.08.025

[bibr48-02698811231158245] Holze F Ley L Müller F , et al. (2022) Direct comparison of the acute effects of lysergic acid diethylamide and psilocybin in a double-blind placebo-controlled study in healthy subjects. Neuropsychopharmacology 47: 1180–1187. 35217796 10.1038/s41386-022-01297-2 PMC9018810

[bibr49-02698811231158245] Hughes RA Heron J Sterne JA , et al. (2019) Accounting for missing data in statistical analyses: Multiple imputation is not always the answer. Int J Epidemiol 48: 1294–1304. 30879056 10.1093/ije/dyz032 PMC6693809

[bibr50-02698811231158245] Huppert FA So TT (2013) Flourishing across Europe: Application of a new conceptual framework for defining well-being. Soc Indic Res 110: 837–861. 23329863 10.1007/s11205-011-9966-7 PMC3545194

[bibr51-02698811231158245] Jakobsen JC Gluud C Wetterslev J , et al. (2017) When and how should multiple imputation be used for handling missing data in randomised clinical trials–a practical guide with flowcharts. BMC Med Res Methodol 17: 162. 29207961 10.1186/s12874-017-0442-1 PMC5717805

[bibr52-02698811231158245] Johnson MW Garcia-Romeu A Cosimano MP , et al. (2014) Pilot study of the 5-HT2AR agonist psilocybin in the treatment of tobacco addiction. J Psychopharmacol 28: 983–992. 25213996 10.1177/0269881114548296 PMC4286320

[bibr53-02698811231158245] Johnson MW Garcia-Romeu A Griffiths RR (2017) Long-term follow-up of psilocybin-facilitated smoking cessation. Am J Drug Alcohol Abuse 43: 55–60. 27441452 10.3109/00952990.2016.1170135 PMC5641975

[bibr54-02698811231158245] Johnson MW Richards WA Griffiths RR (2008) Human hallucinogen research: Guidelines for safety. J Psychopharmacol 22: 603–620. 18593734 10.1177/0269881108093587 PMC3056407

[bibr55-02698811231158245] Karatzias T Hyland P Bradley A , et al. (2019) Risk factors and comorbidity of ICD-11 PTSD and complex PTSD: Findings from a trauma-exposed population based sample of adults in the United Kingdom. Depress Anxiety 36: 887–894. 31268218 10.1002/da.22934

[bibr56-02698811231158245] Khan AJ Bradley E O’Donovan A , et al. (2022) Psilocybin for trauma-related disorders. In: Barrett FS Preller KH (eds) Disruptive Psychopharmacology. Anonymous: Springer, pp. 319–332.

[bibr57-02698811231158245] Kočárová R Horáček J Carhart-Harris R (2021) Does psychedelic therapy have a transdiagnostic action and prophylactic potential? Front Psychiatry 12: 661233. 34349678 10.3389/fpsyt.2021.661233 PMC8327748

[bibr58-02698811231158245] Ko K Knight G Rucker JJ , et al. (2022) Psychedelics, mystical experience, and therapeutic efficacy: A systematic review. Front Psychiatry 13: 917199. 35923458 10.3389/fpsyt.2022.917199 PMC9340494

[bibr59-02698811231158245] Kopra EI Ferris JA Rucker JJ , et al. (2022a) Adverse experiences resulting in emergency medical treatment seeking following the use of lysergic acid diethylamide (LSD). J Psychopharmacol 36: 956–964. 35672900 10.1177/02698811221099650 PMC9353972

[bibr60-02698811231158245] Kopra EI Ferris JA Winstock AR , et al. (2022b) Adverse experiences resulting in emergency medical treatment seeking following the use of magic mushrooms. J Psychopharmacol 36: 965–973. 35388724 10.1177/02698811221084063 PMC9353971

[bibr61-02698811231158245] Krediet E Bostoen T Breeksema J , et al. (2020) Reviewing the potential of psychedelics for the treatment of PTSD. Int J Neuropsychopharmacol 23: 385–400. 32170326 10.1093/ijnp/pyaa018 PMC7311646

[bibr62-02698811231158245] Leger RF Unterwald EM (2022) Assessing the effects of methodological differences on outcomes in the use of psychedelics in the treatment of anxiety and depressive disorders: A systematic review and meta-analysis. J Psychopharmacol 36: 20–30. 34519567 10.1177/02698811211044688

[bibr63-02698811231158245] Leonard JB Anderson B Klein-Schwartz W (2018) Does getting high hurt? Characterization of cases of LSD and psilocybin-containing mushroom exposures to national poison centers between 2000 and 2016. J Psychopharmacol 32: 1286–1294. 30182795 10.1177/0269881118793086

[bibr64-02698811231158245] Mans K Kettner H Haijen ECHM , et al. (2021) Sustained, multifaceted improvements in mental well-being following psychedelic experiences in a prospective opportunity sample. Front Psychiatry 12: 1038. 10.3389/fpsyt.2021.647909 PMC8277190 34267683

[bibr65-02698811231158245] Mason NL Dolder PC Kuypers KP (2020) Reported effects of psychedelic use on those with low well-being given various emotional states and social contexts. Drug Sci Policy Law 6: 2050324519900068.

[bibr66-02698811231158245] Mason NL Kuypers KP (2018) Mental health of a self-selected sample of psychedelic users and self-medication practices with psychedelics. J Psychedelic Stud 2: 45–52.

[bibr67-02698811231158245] Mason NL Mischler E Uthaug MV , et al. (2019) Sub-acute effects of psilocybin on empathy, creative thinking, and subjective well-being. J Psychoactive Drugs 51: 123–134. 30905276 10.1080/02791072.2019.1580804

[bibr68-02698811231158245] Matzopoulos R Morlock R Morlock A , et al. (2021) Psychedelic mushrooms in the USA: Knowledge, patterns of use, and association with health outcomes. Front Psychiatry 12: 780696. 35046855 10.3389/fpsyt.2021.780696 PMC8761614

[bibr69-02698811231158245] McCartney AM McGovern HT De Foe A (2022) Psychedelic assisted therapy for major depressive disorder: Recent work and clinical directions. J Psychedelic Stud 6: 10–22.

[bibr70-02698811231158245] Moreno FA Wiegand CB Taitano EK , et al. (2006) Safety, tolerability, and efficacy of psilocybin in 9 patients with obsessive-compulsive disorder. J Clin Psychiatry 67: 1735–1740. 17196053 10.4088/jcp.v67n1110

[bibr71-02698811231158245] Muller F Kraus E Holze F , et al. (2022) Flashback phenomena after administration of LSD and psilocybin in controlled studies with healthy participants. Psychopharmacology 239: 1933–1943. 35076721 10.1007/s00213-022-06066-z PMC9166883

[bibr72-02698811231158245] Murphy R Kettner HS Zeifman R , et al. (2022) Therapeutic alliance and rapport modulate responses to psilocybin assisted therapy for depression. Front Pharmacol 12: 788155. 10.3389/fphar.2021.788155 PMC9009076 35431912

[bibr73-02698811231158245] Muthukumaraswamy S Forsyth A Lumley T (2021) Blinding and expectancy confounds in psychedelic randomised controlled trials. Expert Rev Clin Pharmacol 14: 1133–1152. 34038314 10.1080/17512433.2021.1933434

[bibr74-02698811231158245] Muthukumaraswamy S Forsyth A Sumner RL (2022) The challenges ahead for psychedelic ‘medicine’. Aust N Z J Psychiatry 56: 1378–1383. 35243919 10.1177/00048674221081763

[bibr75-02698811231158245] Nielson EM May DG Forcehimes AA , et al. (2018) The psychedelic debriefing in alcohol dependence treatment: Illustrating key change phenomena through qualitative content analysis of clinical sessions. Front Pharmacol 9: 132. 29515449 10.3389/fphar.2018.00132 PMC5826346

[bibr76-02698811231158245] Ortiz CE Dourron HM Sweat NW , et al. (2022) Special considerations for evaluating psilocybin-facilitated psychotherapy in vulnerable populations. Neuropharmacology 214: 109127. 35577136 10.1016/j.neuropharm.2022.109127

[bibr77-02698811231158245] Peill JM Trinci KE Kettner H , et al. (2022) Validation of the psychological insight scale: A new scale to assess psychological insight following a psychedelic experience. J Psychopharmacol 36: 31–45. 34983255 10.1177/02698811211066709 PMC8801624

[bibr78-02698811231158245] Pilecki B Luoma JB Bathje GJ , et al. (2021) Ethical and legal issues in psychedelic harm reduction and integration therapy. Harm Reduct J 18: 40. 33827588 10.1186/s12954-021-00489-1 PMC8028769

[bibr79-02698811231158245] Rea K Wallace B (2021) Enhancing equity-oriented care in psychedelic medicine: Utilizing the EQUIP framework. Int J Drug Policy 98: 103429. 34461409 10.1016/j.drugpo.2021.103429

[bibr80-02698811231158245] Rickli A Moning OD Hoener MC , et al. (2016) Receptor interaction profiles of novel psychoactive tryptamines compared with classic hallucinogens. Eur Neuropsychopharmacol 26: 1327–1337. 27216487 10.1016/j.euroneuro.2016.05.001

[bibr81-02698811231158245] Romeo B Hermand M Petillion A , et al. (2021) Clinical and biological predictors of psychedelic response in the treatment of psychiatric and addictive disorders: A systematic review. J Psychiatr Res 137: 273–282. 33730602 10.1016/j.jpsychires.2021.03.002

[bibr82-02698811231158245] Roseman L Nutt DJ Carhart-Harris RL (2018) Quality of acute psychedelic experience predicts therapeutic efficacy of psilocybin for treatment-resistant depression. Front Pharmacol 8: 974. 29387009 10.3389/fphar.2017.00974 PMC5776504

[bibr83-02698811231158245] Rucker JJ Marwood L Ajantaival R-J , et al. (2022) The effects of psilocybin on cognitive and emotional functions in healthy participants: Results from a phase 1, randomised, placebo-controlled trial involving simultaneous psilocybin administration and preparation. J Psychopharmacol 36: 114–125. 35090363 10.1177/02698811211064720 PMC8801675

[bibr84-02698811231158245] Rucker JJ Young AH (2021) Psilocybin: From serendipity to credibility? Front Psychiatry 12: 445. 10.3389/fpsyt.2021.659044 PMC8096916 33967860

[bibr85-02698811231158245] Rucker JJH Jelen LA Flynn S , et al. (2016) Psychedelics in the treatment of unipolar mood disorders: A systematic review. J Psychopharmacol 30: 1220–1229. 27856684 10.1177/0269881116679368

[bibr86-02698811231158245] Sepeda ND Clifton JM Doyle LY , et al. (2020) Inhaled 5-methoxy-N, N-dimethyltryptamine: Supportive context associated with positive acute and enduring effects. J Psychedelic Stud 4: 114–122.

[bibr87-02698811231158245] Sessa B Fischer FM (2015) Underground MDMA-, LSD-and 2-CB-assisted individual and group psychotherapy in Zurich: Outcomes, implications and commentary. Drug Sci Policy Law 2: 2050324515578080.

[bibr88-02698811231158245] Shaw L Rea K Lachowsky NJ , et al. (2022) Magic mushroom use: A qualitative interview study of post-trip impacts and strategies for optimizing experiences. J Psychoactive Drugs. Epub ahead of print 22 March 2022. DOI: 10.1080/02791072.2022.2054746. 35315749

[bibr89-02698811231158245] Soares CM Leite Â Pinto M (2022) Self-care practices with psychedelics–a qualitative study of users’ perspectives. J Psychoactive Drugs. Epub ahead of print 15 May 2022. DOI: 10.1080/02791072.2022.2071134. 35574941

[bibr90-02698811231158245] Spriggs MJ Kettner H Carhart-Harris RL (2021) Positive effects of psychedelics on depression and wellbeing scores in individuals reporting an eating disorder. Eat Weight Disord 26: 1265–1270. 32895801 10.1007/s40519-020-01000-8

[bibr91-02698811231158245] St Arnaud KO Sharpe D (2022) Contextual parameters associated with positive and negative mental health in recreational psychedelic users. J Psychoactive Drugs. Epub ahead of print 13 February 2022. DOI: 10.1080/02791072.2022.2039815. 35156542

[bibr92-02698811231158245] Studerus E Gamma A Kometer M , et al. (2012) Prediction of psilocybin response in healthy volunteers. PLoS One 7: e30800. 10.1371/journal.pone.0030800 PMC3281871 22363492

[bibr93-02698811231158245] Studerus E Kometer M Hasler F , et al. (2011) Acute, subacute and long-term subjective effects of psilocybin in healthy humans: A pooled analysis of experimental studies. J Psychopharmacol 25: 1434–1452. 20855349 10.1177/0269881110382466

[bibr94-02698811231158245] Tavakol M Dennick R (2011) Making sense of Cronbach’s alpha. Int J Med Educ 2: 53. 28029643 10.5116/ijme.4dfb.8dfd PMC4205511

[bibr95-02698811231158245] Timmermann C Watts R Dupuis D (2020) Towards psychedelic apprenticeship: Developing a gentle touch for the mediation and validation of psychedelic-induced insights and revelations. Transcult Psychiatry 59: 691–704. 10.1177/13634615221082796 PMC9660279 35313754

[bibr96-02698811231158245] Watts R Day C Krzanowski J , et al. (2017) Patients’ Accounts of Increased “Connectedness” and “Acceptance” After Psilocybin for Treatment-Resistant Depression. Los Angeles, CA: Sage Publications.

[bibr97-02698811231158245] Weston NM Gibbs D Bird CI , et al. (2020) Historic psychedelic drug trials and the treatment of anxiety disorders. Depress Anxiety 37: 1261–1279. 32627308 10.1002/da.23065

[bibr98-02698811231158245] Williams MT Davis AK Xin Y , et al. (2021) People of color in North America report improvements in racial trauma and mental health symptoms following psychedelic experiences. Drugs 28: 215–226. 34349358 10.1080/09687637.2020.1854688 PMC8330400

[bibr99-02698811231158245] Winstock AR Davies EL Ferris JA , et al. (2022) Using the Global Drug Survey for Harm Reduction, vol. 2. Lisbon: European Monitoring Centre for Drugs and Drug Addiction (EMCDDA).

[bibr100-02698811231158245] Winstock AR Timmerman C Davies E , et al. (2021) Global Drug Survey (GDS) 2020 psychedelics key findings report. Global Drug Survey.

[bibr101-02698811231158245] Yaden DB Griffiths RR (2020) The subjective effects of psychedelics are necessary for their enduring therapeutic effects. ACS Pharmacol Transl Sci 4: 568–572. 33861219 10.1021/acsptsci.0c00194 PMC8033615

[bibr102-02698811231158245] Yockey RA Vidourek RA King KA (2020) Trends in LSD use among US adults: 2015–2018. Drug Alcohol Depend 212: 108071. 32450479 10.1016/j.drugalcdep.2020.108071

[bibr103-02698811231158245] Zayfert C Dums AR Ferguson RJ , et al. (2002) Health functioning impairments associated with posttraumatic stress disorder, anxiety disorders, and depression. J Nerv Ment Dis 190: 233–240. 11960084 10.1097/00005053-200204000-00004

[bibr104-02698811231158245] Zeifman RJ Wagner AC Watts R , et al. (2020) Post-psychedelic reductions in experiential avoidance are associated with decreases in depression severity and suicidal ideation. Front Psychiatry 11: 782. 32903724 10.3389/fpsyt.2020.00782 PMC7438781

