# Supplementary material for: Investigation of self-treatment with lysergic acid diethylamide and psilocybin mushrooms: Findings from the Global Drug Survey 2020
Source: J Psychopharmacol. 2023 Mar 6;37(7):733–48. doi: 10.1177/02698811231158245 (PMC10350727; doi:10.1177/02698811231158245)

**Supplementary Table S1.** Conditions and problems treated

|  | **Frequency** | **Valid %** |
| --- | --- | --- |
| Depression | 1,338 | 40.2 |
| Anxiety | 666 | 20.0 |
| Relationship problem | 308 | 9.3 |
| Trauma | 164 | 4.9 |
| Alcohol or other substance use disorder | 152 | 4.6 |
| PTSD | 118 | 3.5 |
| Bipolar | 66 | 2.0 |
| Bereavement | 63 | 1.9 |
| Distress associated with a medical condition/diagnosis | 48 | 1.4 |
| Cancer-related distress | 4 | 0.1 |
| Mental health distress associated to cancer diagnosis | 4 | 0.1 |
| OCD | 27 | 0.8 |
| Anorexia/Bulimia | 23 | 0.7 |
| Psychosis | 19 | 0.6 |
| Other | 335 | 10.1 |
| Borderline Personality Disorder | 15 | 0.5 |
| ADHD | 12 | 0.4 |
| Overeating (Obesity) | 11 | 0.3 |
| Chronic pain | 10 | 0.3 |
| To increase appetite | 3 | 0.1 |
| [Missing] | [37] |  |
| Total | 3,327 | 100.0 |

Frequencies of primary conditions or problems respondents indicated self-treating. For analyses purposes, the initial 19 categories were reduced to 13 due to low counts (below 0.5% of respondents) in 6 categories. ‘Cancer-related distress’ and ‘Mental health distress associated to cancer diagnosis’ were grouped together with ‘Distress associated with another medical disorder’, and the new category renamed ‘Distress associated with a medical condition/diagnosis’. ‘Overeating (Obesity)’, ‘Chronic pain’, and ‘To increase appetite’ were grouped with the original category ‘Other’. One original category ‘Treating cancer itself’ had a count of zero.

**Supplementary Methods.** Full response items for question: “How would you describe the maximal short-term effects of the dose you usually took for therapeutic purposes?”

- Intense psychedelic experience (+/- mystical / spiritual elements) with marked emotional / spiritual exploration with marked hallucinations, perceptual / cognitive distortions.

- Moderate psychedelic experience with enhancement of environments and social interaction with some perceptual / cognitive distortion, social interaction and enjoyment of but not interfering significantly with social interaction.
- Mild psychedelic experience - predominately seeking increase in mood and energy but little in the way of perceptual and thought disorder.
- No psychedelic experience, but other acute effects.
- No experience or effects at all

**Supplementary Table S2.** Demographics of respondents self-treating with LSD or psilocybin

|  | **N** | **Valid %** |
| --- | --- | --- |
| **Age** | 3,364 |  |
| 16-19 | 891 | 26.5 |
| 20-24 | 1,074 | 31.9 |
| 25-34 | 949 | 28.2 |
| 35+ | 450 | 13.4 |
| **Gender** | 3,364 |  |
| Male | 2,422 | 72.0 |
| Female | 825 | 24.5 |
| Non-binary | 88 | 2.6 |
| Different identity | 29 | 0.9 |
| **Country of Residence** | 3,364 |  |
| U.S | 865 | 25.7 |
| Germany | 341 | 10.1 |
| Australia | 295 | 8.8 |
| Finland | 254 | 7.6 |
| England | 172 | 5.1 |
| Canada | 164 | 4.9 |
| Brazil | 148 | 4.4 |
| Other | 1,125 | 33.4 |
| **Ethnicity** | 3,337 |  |
| White | 2,679 | 80.3 |
| Hispanic / Latino | 262 | 7.9 |
| Mixed | 208 | 6.2 |
| Other | 188 | 5.6 |
| [Missing] | [27] |  |
| **Highest Education** | 3,263 |  |
| Primary school or no formal schooling | 272 | 8.3 |
| Lower secondary school | 333 | 10.2 |
| Higher/Secondary Education | 1,168 | 35.8 |
| College certificate / diploma | 587 | 18.0 |
| UG degree | 665 | 20.4 |
| PG degree | 238 | 7.3 |
| [Missing] | [101] |  |
| **Lifetime mental health diagnosis** | 3,360 |  |
| None | 1,461 | 43.5 |
| Yes***** | 1,899 | 56.5 |
| Depression | 1,495 | 44.5 |
| Anxiety | 1,243 | 37.0 |
| ADHD | 530 | 15.8 |
| PTSD | 459 | 10.7 |
| Bipolar | 230 | 6.8 |
| Psychosis | 108 | 3.2 |
| Other | 356 | 10.6 |
| [Missing] | [4] |  |
| **Prescribed medication for mental health condition†** | 3,329 |  |
| Yes (currently) | 702 | 21.1 |
| Yes (in the past) | 793 | 23.8 |
| No (not prescribed) | 373 | 11.2 |
| No (no diagnosed condition)**†** | 1,461 | 43.8 |
| [Missing] | [31] |  |

*Able to select more than one condition, hence percentages of conditions add up to above 100%

†Question only asked from those with one or more lifetime mental health diagnoses; those without diagnoses added to the denominator, for percentages to reflect prevalence of medications in the whole sample

**Supplementary Table S3.** Sensitivity analysis of OLS Regression among respondents with max. 5 missing values or N/As

|  | Unst. Coeff. | | Std. Coeff. | t | Sig. | 99.8% Cl for B | |
| --- | --- | --- | --- | --- | --- | --- | --- |
|  | B | SE | β |  |  | Lower Bound | Upper Bound |
| (Constant) | 1.395 | 0.063 |  | 22.004***** | <0.001 | 1.199 | 1.591 |
| Age | 0.001 | 0.002 | 0.009 | 0.473 | 0.636 | -0.005 | 0.006 |
| Gender |  |  |  |  |  |  |  |
| Female | -0.022 | 0.034 | -0.012 | -0.670 | 0.503 | -0.126 | 0.081 |
| Non-binary OR Other | 0.048 | 0.075 | 0.012 | 0.651 | 0.515 | -0.182 | 0.279 |
| Male | Ref |  |  |  |  |  |  |
| Substance |  |  |  |  |  |  |  |
| LSD | -0.118 | 0.029 | -0.075 | -4.061***** | <0.001 | -0.208 | -0.028 |
| Psilocybin Mushrooms | Ref |  |  |  |  |  |  |
| Seeking Advice |  |  |  |  |  |  |  |
| Yes | 0.214 | 0.036 | 0.108 | 5.985***** | <0.001 | 0.103 | 0.324 |
| No | Ref |  |  |  |  |  |  |
| Intensity |  |  |  |  |  |  |  |
| No experience or effects at all | -0.954 | 0.175 | -0.098 | -5.436***** | <0.001 | -1.497 | -0.411 |
| No psychedelic experience, but other acute effects | -0.245 | 0.084 | -0.053 | -2.906 | 0.004 | -0.505 | 0.016 |
| Mild psych experience | -0.205 | 0.047 | -0.084 | -4.389***** | <0.001 | -0.350 | -0.061 |
| Moderate psych experience | -0.224 | 0.031 | -0.138 | -7.272***** | <0.001 | -0.319 | -0.128 |
| Intense psych experience | Ref |  |  |  |  |  |  |
| Primary condition treated |  |  |  |  |  |  |  |
| Anxiety | 0.054 | 0.038 | 0.028 | 1.429 | 0.153 | -0.063 | 0.172 |
| OCD | -0.231 | 0.175 | -0.024 | -1.321 | 0.187 | -0.771 | 0.309 |
| Bipolar | 0.061 | 0.101 | 0.011 | 0.607 | 0.544 | -0.251 | 0.374 |
| PTSD | 0.334 | 0.076 | 0.081 | 4.382***** | <0.001 | 0.098 | 0.569 |
| Psychosis | 0.007 | 0.190 | 0.001 | 0.036 | 0.972 | -0.582 | 0.595 |
| Alcohol or other SUD | -0.001 | 0.069 | 0.000 | -0.007 | 0.994 | -0.215 | 0.214 |
| Anorexia/Bulimia | -0.543 | 0.176 | -0.056 | -3.090 | 0.002 | -1.086 | 0.001 |
| Distress associated with a medical condition/diagnosis | -0.173 | 0.120 | -0.026 | -1.439 | 0.150 | -0.545 | 0.199 |
| Bereavement | -0.242 | 0.104 | -0.043 | -2.331 | 0.020 | -0.562 | 0.079 |
| Trauma | 0.037 | 0.066 | 0.010 | 0.559 | 0.576 | -0.168 | 0.241 |
| Relationship problem | -0.085 | 0.052 | -0.031 | -1.637 | 0.102 | -0.247 | 0.076 |
| Other | 0.104 | 0.049 | 0.040 | 2.111 | 0.035 | -0.049 | 0.258 |
| Depression | Ref |  |  |  |  |  |  |

Dependent Variable: 17-item Outcome Scale. Unst. = unstandardized; Coeff. = coefficient; Std. = Standardized; SE = standard error; Ref = reference category. * = p<.002. Model based on 2,935 complete cases.

**Supplementary Figure S1**. Predicted probability of each level of experience intensity at each level of duration of positive outcomes


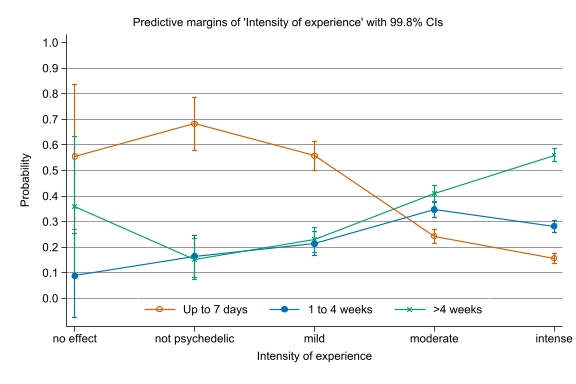

Supplement: sj-docx-1-jop-10.1177_02698811231158245 – Supplemental material for Investigation of self-treatment with lysergic acid diethylamide and psilocybin mushrooms: Findings from the Global Drug Survey 2020 [file sj-docx-1-jop-10.1177_02698811231158245.docx]
